# Lateralized Movements during the Mating Behavior, Which Are Associated with Sex and Sexual Experience, Increase the Mating Success in *Alphitobius diaperinus* (Coleoptera: Tenebrionidae)

**DOI:** 10.3390/insects14100806

**Published:** 2023-10-10

**Authors:** Erika Calla-Quispe, Esperanza Irigoin, Madina Mansurova, Carlos Martel, Alfredo J. Ibáñez

**Affiliations:** 1 Instituto de Ciencias Ómicas y Biotecnología Aplicada, Pontificia Universidad Católica del Perú, Av. Universitaria 1801, San Miguel, Lima 15088, Peru; esperanzairigoin.23@gmail.com (E.I.); mmansurova@pucp.edu.pe (M.M.); aibanez@pucp.edu.pe (A.J.I.); 2 Escuela de Biología, Facultad de Ciencias Biológicas, Universidad Nacional Pedro Ruiz Gallo, Calle Juan XXIII 391, Lambayeque 14013, Peru; 3 Trait Diversity and Function, Royal Botanic Gardens, Kew, Richmond TW9 3AB, UK; 4 Departamento de Ciencias, Pontificia Universidad Católica del Perú, Av. Universitaria 1801, San Miguel, Lima 15088, Peru

**Keywords:** *Alphitobius diaperinus*, lateralization, asymmetric behavior, mating behavior, sexual experience, beetle learning, poultry pest

## Abstract

**Simple Summary:**

Lateralization in mating behavior seems to be a common feature among insects, including some pest species. The poultry pest, *Alphitobius diaperinus*, has a mating behavior and success that is affected by the interplay between its sexual identity and sexual experience. Although it is expected that the directionality of the displacement (i.e., lateralization and non-lateralization) during the mating display affects the mating success in *A. diaperinus*, it is unknown whether this is the case. In the present study, we evaluated how lateralized and non-lateralized movements of *A. diaperinus* adults affect mate success in relation to the sex and sexual experience of the mating pairs. The study highlights the impact of lateralization in mating behavior and the superior skills of experienced males to achieve mating success, which seems to be related to beetle learning. Our study addresses the importance of learning in improving beetle fitness and contributes to the growing understanding of lateralized behavior on insect mating behavior. This finding enhances our comprehension of these pests, potentially contributing to the eventual mitigation of their infestation within the poultry facility.

**Abstract:**

In the present study, we explored the effects of displacement directionality in mating behavior (i.e., lateralized and non-lateralized movements) on mating success (i.e., copulation occurs) and efficiency (i.e., time length at which copulation is achieved), and its association with sex and sexual experience in *A. diaperinus*. To do so, we carried out mating experiments and recorded the behavior of the mating pair during the whole mating sequence (i.e., precopulatory and copulatory phases). During the precopulatory phase, independently of sex and sexual experience, all beetles performed non-lateralized (i.e., backside or frontside) approaches; however, only sexually experienced beetles showed lateralized approaches (i.e., right-side and left-side). Notably, experienced males exhibited greater mating success than virgin males. After the approach, both virgin and experienced males displayed lateralized and non-lateralized mounts on the females with distinct mating success. Regardless of their sexual experience, 100% of successful mating attempts were achieved when males mounted from the females’ right side. Furthermore, the development of lateralized approaches and mounts reduces the time of mating sequence span compared with non-lateralized behaviors. We highlight the importance of lateralization in mating behavior and sexual experience to achieve higher mating success, addressing a potential learning ability of beetles based on experience.

## 1. Introduction

Lateralization of the brain (i.e., the division of functions and/or structures between the left and right hemispheres) is a common phenomenon in many animal species. This specialization of brain function confers a dominance in movement directionality during the development of certain behaviors and, thus, is the cause of the asymmetric behaviors developed by animals (e.g., the dominance in the use of the right or left hand in humans). In insects, this can be observed during their different activities, but it is pronounced during the mating phases (i.e., approaching and mounting) when partners develop premating and mating behavior by performing lateralized (i.e., movements to their right or left side) and non-lateralized movements (i.e., movements to their back or front side). These differences in directionality can affect mating success and therefore have significant functional advantages that contribute to biological fitness [1]. Even though diverse insects have shown preferences for one side of the mate’s body or a tendency to turn in a particular direction during mating (i.e., mating behavioral asymmetries; [2,3,4,5]), particular attention has been focused on beetle pests [6,7,8,9,10,11,12,13] since it may help to understand their success and explosive reproduction.

Premating and mating behaviors in insects are influenced by a range of factors (e.g., age, sex, sexual experience [6,8,14]), which impact the mating success. In this context, lateralized behavior during the approaching and mounting of the mating pair may confer advantages in increasing the efficiency, accuracy, or speed in mating, while non-lateralized behavior may allow for quicker and more flexible responses to changing environmental conditions [1,15]. For instance, in some beetle pest species such as *Cryptolestes ferrugineus*, *Tribolium castaneum*, *T. confusum* and *Trogoderma granarium*, males with left-biased approaches had a higher mating success than ones with right-biased approaches or non-lateralized behavior [8,9,10,11,12,13]. However, the advantages of these behaviors are context-dependent (e.g., habitat, environmental condition) and associated with intra-specific characteristics such as the mating system and habitat specialization as recorded in *Rhyzoperha dominica*, *Sitophilus oryzae* and *Tenebrio molitor* beetle pests [6,7,10,16].

The beetle pest *Alphitobius diaperinus* (Coleoptera: Tenebrionidae), also known as the lesser mealworm, is a widespread pest that primarily affects the poultry industry by causing significant structural damage to poultry houses and facilitating the transmission of avian pathogens [17,18,19,20]. The species has adapted to the moist, temperature-controlled environments of poultry production facilities [17,18,19,20]. Although this species might have originated in sub-Saharan Africa, it is currently widely distributed worldwide, and there are reports of infestations from all the continents [18]. In Peru, *A. diaperinus* is also commonly recorded infesting poultry houses, despite the species having been reported for the first time in the country only three decades ago by Vergara and Gazani [21]. However, how and when precisely the pest was introduced in Peru remains unknown [21]. The rapid spread of this pest can be related to the reproductive characteristics as well as their insecticide resistance and long-life span, which can last up to one year as adults [17,18].

Previous research has shown that adults of both *A. diaperinus* sexes, independently of their sexual experience, are attracted to and release generalized long-range attractive chemical signals. However, sexual experience does affect the mating responses to short-range chemical signals in *A. diaperinus* adults, with stronger and longer mating responses exhibited by virgin males compared to sexually experienced males [14]. Since the behaviors displayed by experienced males seemed to be distinct but also more efficient (i.e., shorter premating and mating display until copulation occurs) from those of virgin males (Calla-Quispe, pers. obs.), potential behavioral asymmetries in the mating behavior (i.e., lateralized and non-lateralized movements) may be associated with changes in sexual experience and the sex. However, whether these happen or not in *A. diaperinus* has not yet been explored. This study, aimed to investigate the occurrence and role of lateralized and non-lateralized mating behavior on mating success and efficiency, and their association with sex and sexual experience in *A. diaperinus*. In order to do this, we conducted mating behavioral experiments with adult beetles, measured the magnitude of asymmetric mating behaviors, which were depicted in an ethogram, and carried out statistical tests.

## 2. Materials and Methods

### 2.1. Studied Species and Culture in the Laboratory

Adults of *A. diaperinus* were obtained from a commercial poultry production facility located in the surroundings of Lima (12°09′27.8″ S 76°53′46.2″ W) and transported to the laboratory for breeding. Beetles were bred within a glass box (30 cm × 25 cm × 20 cm) placed in an environmentally controlled climate chamber (Memmert HPP750, Memmert GmbH, Schwabach, Germany), which was set up at 30 °C, 50% relative humidity and a photoperiod of 12:12 h (light–dark). Individuals of *A. diaperinus* were reared on wheat flour and tap water, which was provided using a humidifying towel paper. The second generation of larvae was extracted from the main glass box and kept in groups of 10 individuals in Petri dishes (60 mm diam. × 15 mm height). Some of the pupae were then transferred to another glass box, whereas the remaining pupae were individually isolated in Petri dishes. Sex identification was carried out during the pupa phase (i.e., male “M” and female “F”). Adult beetles in the glass box were free to mate and reproduce (experienced “exp”), whereas those isolated in Petri dishes remained without sexual contact (virgins “v”).

### 2.2. Experiments on the Role of Asymmetric Mating Behaviors on Mating Success

**Figure 1:**
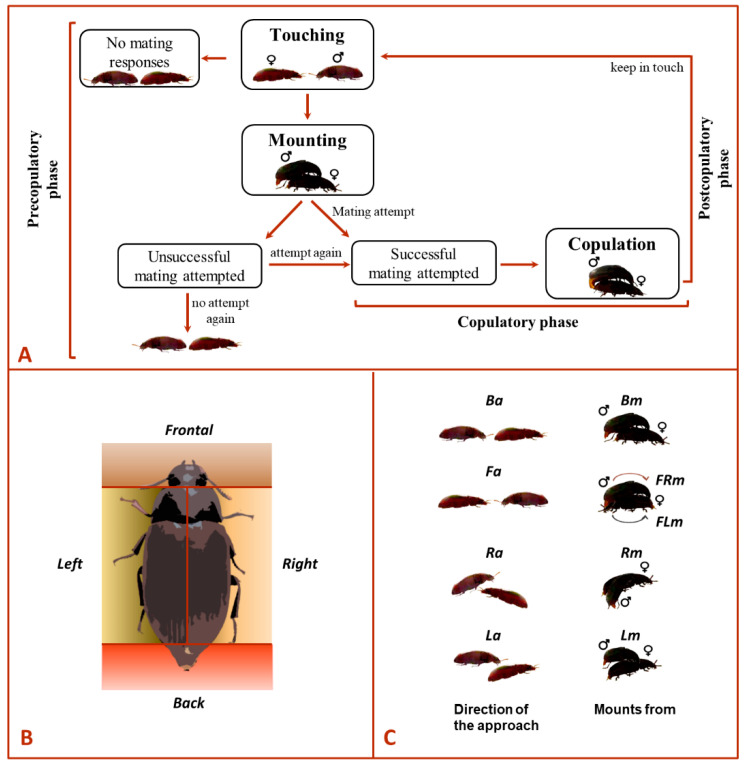
Ethogram depicting (**A**) the sequence of mating behavior, (**B**) graphical representation of the side chosen by the mate and (**C**) types of movements for approaching (*Ba* backside, *Fa* frontside, *Ra* right-side, *La* left-side) and mounting (*Bm* backside, *FRm* front-right, *FLm* front-left, *Rm* right-side, *Lm* left-side) in *Alphitobius diaperinus*.

For the experiments, four different groups were implemented based on the beetle sex and its sexual experience, i.e., Mv–Fv, Mv–Fexp, Mexp–Fv and Mexp–Fexp. For all bioassays involving virgin beetles, they were 21-days-old or older to guarantee sexual maturity. All tested beetles were used only once. Prior to carrying out each experiment, males and females were individually removed from the climate chamber and left to acclimate for one hour to laboratory light and temperature conditions. Then, males and females were transferred to a Petri dish arena (60 mm diam. × 15 mm height) containing a filter paper at the inner bottom and the Petri walls to surround the experimental arena in order to limit the exposition of visual cues, which might impact the behavior of the tested beetles during the bioassays [10]. The ethogram in Figure 1A describes the mating behavioral sequence in *A. diaperinus*, from the precopulatory to the postcopulatory phase. Each male and female was placed in the extremes of the arena facing each other. Then, the behavior of the couple was observed and documented for 10 min with the help of video camcorders. We specifically recorded the sex of the beetle that approached the potential mate (i.e., only the male approached, only the female approached, or both simultaneously approached each other), the mate’s side approached by the individual (i.e., first contact made with the mate from its right-side [Ra], left-side [La], backside [Ba], or frontside [Fa]; Figure 1B indicates the areas considered for each classified side), the male’s directionality (i.e., female’s left-side, right-side, backside or frontside) during mounting and the side chosen by the male (i.e., mounting the female from its right-side [Rm], left-side [Lm], backside [Bm], front-right [FRm] or front-left [FLm]; see Figure 1C) to attempt copulation were recorded. We also recorded the duration of each mating phase: (i) touching (i.e., individual contacting with their antennae or prothoracic leg), (ii) mounting (i.e., the male is over the female and exposes his genitalia), (iii) copulation (i.e., male inserted the genitalia into the female sexual organ) and (iv) duration of the post-copula interaction (i.e., beetles contacting each other with their antennae or prothoracic leg). Finally, we recorded the number of successful (i.e., copulation occurred) and unsuccessful mating attempts (i.e., no copulation occurred) in each experiment trial.

Although a total of 63 trials were carried out, those without interactions between males and females were discarded. Thus, 58 experimental trials (Mv–Fv: *n* = 13, Mexp–Fv: *n* = 13, Mv–Fexp: *n* = 16, Mexp–Fexp: *n* = 16) were accounted for in the flow chart analyses. For the quantitative analysis of the effect of lateralization on mating success, each entire sequence of mating attempts (i.e., approaching + touching + mounting + (no) copulation) was considered as a single event within each trial since a mating pair could try to mate more than once per trial and could display a different mating behavior.

### 2.3. Statistical Analysis

To test for differences about which sex performed the approach to its mate, the proportion of individuals who initiated the approach was compared using the chi-square (χ^2^) test with Yates’ correction (*p* ≤ 0.05).

To evaluate the effect of the beetle sex that performed the approach and the direction of the approach on mating success, we used a generalized linear model with binomial distribution and a logit link function as described below:

(1) $$\gamma_{1}=MP+IA+DA+\varepsilon_{1}$$

where $\gamma_{1}$ is the vector of the observations (i.e., successful or not successful mating), *MP* is the mating pair combination, *IA* is the sex who performed the approach, *DA* is the direction of the approach and $\varepsilon_{1}$ is the vector of the random residual effect.

To evaluate the effect of the directionality of the male mounting on mating success, we also used a generalized linear model with binomial distribution and a logit link function as described below:

(2) $$\gamma_{2}=MP+DM+\varepsilon_{2}$$

where $\gamma_{2}$ is the vector of the observations (i.e., successful or not successful mating), *MP* is the mating pair, *DM* is the direction of mounting and $\varepsilon_{2}$ is the vector of the random residual effect. In this case, three scenarios were evaluated independently: when the approach was developed by (i) males, (ii) females, or (iii) both sexes simultaneously.

To identify the effect of the directionality during mating behavior on the span of touching and mounting, and its association with sex and sexual experience, we implemented two log-truncated linear models, one having touching span and the other mounting span as response variables; the directionalities of the mating behavior and mating pair combination were set as the explanatory variables. Bonferroni post hoc tests were then conducted. The touching and mounting span were log-transformed before the analyses.

All statistical analyses were carried out using the *stats* [22] and *emmeans* packages [23] in the software R, version 4.1.2 [22].

## 3. Results

### 3.1. Mating Behavior

**Figure 2:**
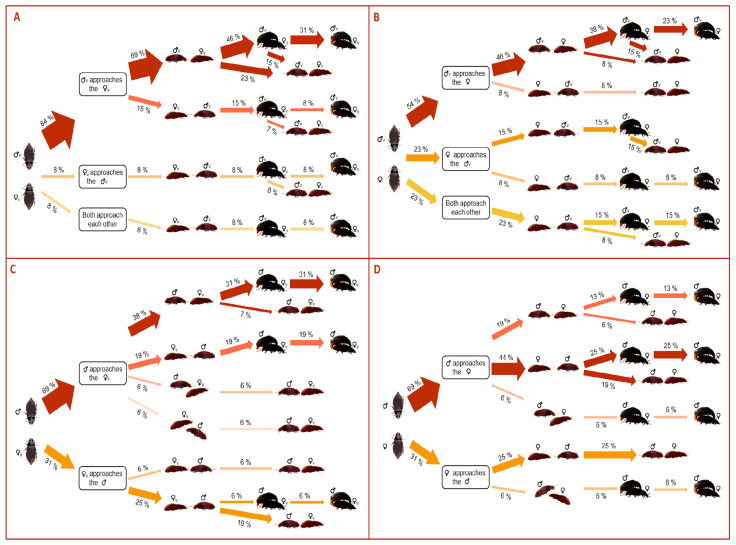
Flow chart quantifying the proportion in the approaching directionality among the pair mating combinations of *Alphitobius diaperinus*: (**A**) Mv–Fv, (**B**) Mv–Fexp, (**C**) Mexp–Fv, (**D**) Mexp–Fexp. The thickness of the arrows indicates the proportion of beetles displaying each behavior.

**Figure 3:**
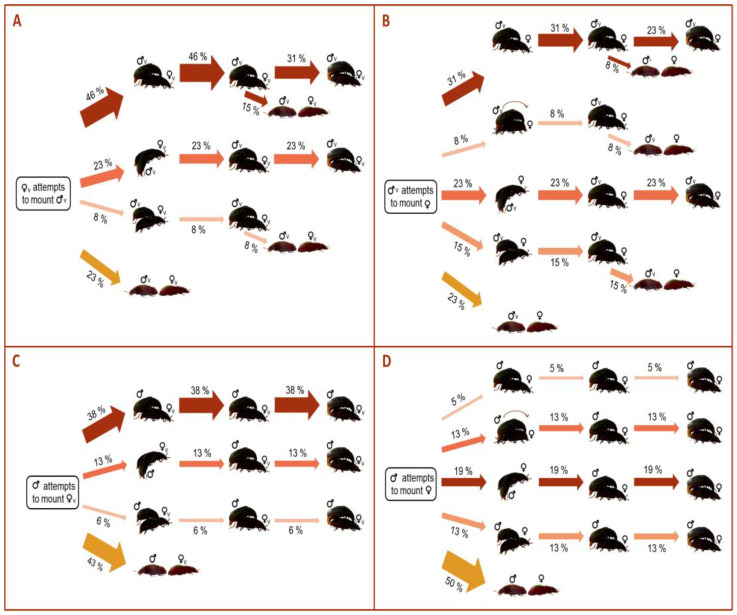
Flow chart quantifying the proportion in the directionality of the mounting among the pair mating combinations of *Alphitobius diaperinus*: (**A**) Mv–Fv, (**B**) Mv–Fexp, (**C**) Mexp–Fv, (**D**) Mexp–Fexp. The thickness of each arrow indicates the proportion of beetles displaying each behavior.

The precopulatory phase begins when one or both beetles of the mating pair perform the lateralized or non-lateralized approach to the mate. After approaching, beetles physically contacted each other’s head, pronotum and elytra with their antennae, maxillary palp and prothoracic legs. Males showed two observable behaviors; in some cases, they exposed their genitalia while walking before approaching the female, and in others, they lifted the female from the female’s abdomen tip to touch the metathorax and sternites. Once the male recognized the female by rearing and antennal waving, the male displayed lateralized or non-lateralized behavior to mount and climb on the female’s back. It is in this phase that the male aligns the body in the same direction as the female (Figure 2 and Figure 3). The male then raised upwards the abdomen tip and exposed their genitalia, whereas the female opened the terminal abdominal segments and intermittently exposed their genitalia. During this phase, males touch the female’s cuticle with their antennae, maxillary palp, and prothoracic and mesothoracic legs. Some Fexp even positioned themselves under the males and then exposed the last sternite to facilitate the mating. In the copulatory phase, the male grasped the female’s thorax with his prothoracic and mesothoracic legs and inserted his genitalia into the female’s sexual organ, while the female slightly raised upwards the abdomen tip. Copulation events occurred 2.5 times per trial, independently of the mating pair. After mating, in the post-copulatory phase, both adults touched each other’s head, pronotum and elytra with their antennae, maxillary palp and prothoracic legs; in addition, males touched the female’s ventral abdomen with their antennae and maxillary palp.

### 3.2. Mating Behavior Display

The approaching behavior of the mating pairs of *A. diaperinus* depends on the beetle sex performing the approach (χ^2^ = 48.2, *d.f.* = 6, *p* < 0.0001). Males predominantly performed the approach to the mate compared to females (84%, 54%, 69%, 69% of the times in Mv–Fv, Mv–Fexp, Mexp–Fv, Mexp–Fexp, Figure 2). In the combinations involving Mv (i.e., Mv–Fv and Mv–Fexp), males only developed non-lateralized behavior in which they predominantly approached from the females’ back (69% and 46% in Mv–Fv and Mv–Fexp, respectively, Figure 2). However, in pairs involving Mexp, males occasionally developed lateralized approaches (12% and 6% in Mexp–Fv and Mexp–Fexp, Figure 2). Although females rarely performed the approach, when they did, the behavior was similar to that of males; only Fexp developed lateralized behaviors (Figure 2). Whereas more than half of the non-lateralized approaches usually ended up in mating, lateralized approaches always ended up in copulation (Figure 2).

After the partners approached, a higher proportion of Mv proceeded to mount the females compared to Mexp (77%, 77%, 57% and 50% in Mv–Fv, Mv–Fexp, Mexp–Fv and Mexp–Fexp, respectively) (Figure 3). Interestingly, when the pair combination did not involve two experienced adults (i.e., Mexp–Fexp), mounts were predominantly performed from the female’s back (46%, 31%, 38% of the times in Mv–Fv, Mv–Fexp, and Mexp–Fv, respectively, Figure 3). However, lateralized behaviors (32% of the time) predominate over non-lateralized ones (18% of the time) in the Mexp–Fexp combination (Figure 3). Moreover, no mounting from the front was recorded in mating pairs involving Fv (Figure 3). Although a lower proportion of mounts was performed in the mating pairs involving Mexp compared with those involving Mv, all the mounts performed by Mexp ended up in copulation, whereas this was not the case for Mv (Figure 3).

### 3.3. Effect Approach and Mounting on Mating Success

**Figure 4:**
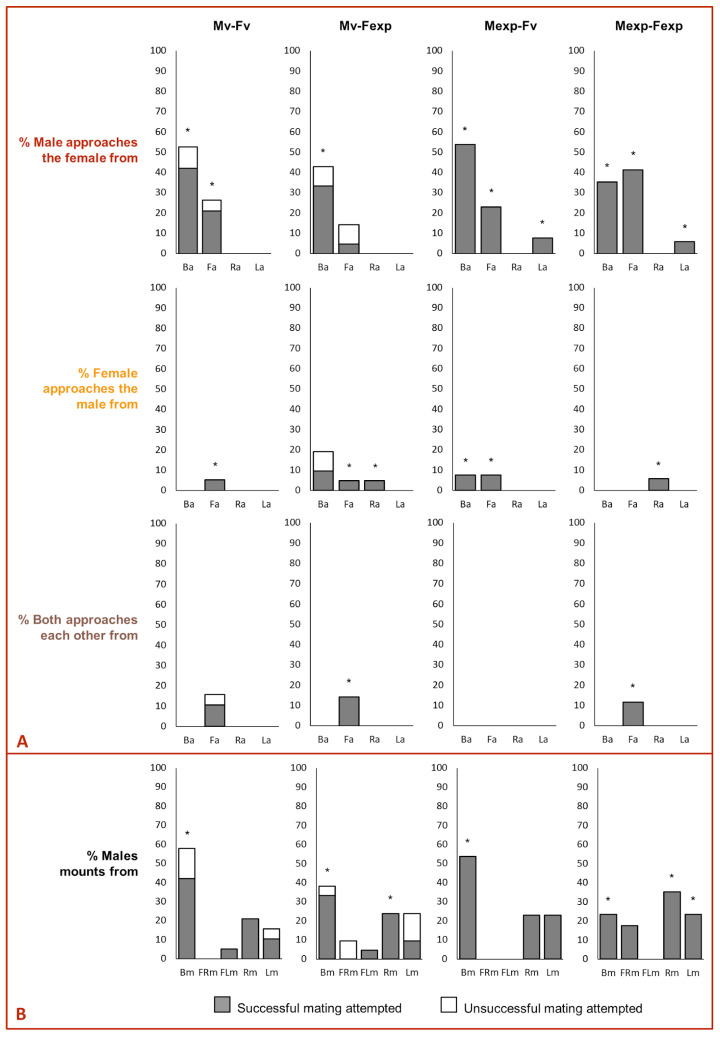
Impact of (**A**) beetle sex performing asymmetric approach behavior and (**B**) male performing asymmetric mounting behavior on mating success of *Alphitobius diaperinus*. Asterisks indicate a significant difference between successful and unsuccessful mating attempts (General linear model, Tukey’s HSD test, *p* < 0.05).

The mating success in the experiments was not affected by the direction of the approach (χ^2^ = 0.27, *d.f.* = 64, *p* = 0.8726, Figure 4A). However, it was affected by the beetle pair combination (χ^2^ = 12.7, *d.f.* = 66, *p* = 0.0053, Figure 4A). Mating success was significantly higher in Mexp–Fexp compared to Mv–Fexp (Tukey’s post hoc test, *p* = 0.05), and there was no other statistical difference when comparing the remaining combinations (Tukey’s post hoc test, for all *p* > 0.05). Mating success was affected by the combined effect of the approaching sex and the direction of approach (χ^2^ = 72.1, *d.f.* = 51, *p* < 0.0001, Figure 4A), as well as the combined effect of mating pair combination, the approaching sex the approach and the direction of the approach (χ^2^ = 681.9, *d.f.* = 49, *p* < 0.0001, Figure 4A). Regardless of the beetle pair combination and the approaching sex, the mating attempt (i.e., the male exposes its genitalia and tries to penetrate the female) was always successful when a lateralized approach was displayed followed by a mount.

When the males performed the approach, the mating success was affected by the beetle pair combination (χ^2^ = 12.1, *d.f.* = 47, *p* = 0.0072) but not by the direction of mounting (χ^2^ = 0.1, *d.f.* = 50, *p* = 0.7115, Figure 4B). Mating success was shorter in Mv–Fexp compared to Mv–Fv, Mexp–Fv and Mexp–Fexp (for all *p* < 0.05). In contrast, when only the females performed the approach, the mating success was not affected by the beetle pair combination (χ^2^ = 2.3, *d.f.* = 5, *p* = 0.5145) or the direction of mounting (χ^2^ = 0.4, *d.f.* = 8, *p* = 0.5036). However, when both sexes performed the approach simultaneously, the mating success was affected by the direction of mounting (χ^2^ = 6.0, *d.f.* = 6, *p* = 0.0141), but not by the mating pair combination (χ^2^ = 0.0, *d.f.* = 4, *p* = 1.000). Males mounting from the right-side were significantly more successful than those mounting from the left-side or backside (for all *p* < 0.05). Regardless of the beetle pair combination and which sex performed the approach, the mating attempt was always successful when the male displayed right-side or front-left mounting over the female (Figure 4B).

### 3.4. Effect of the Mating Pair, the Direction of Approaching and Mounting on the Interaction Time

In our experiments, we did not find differences in time for the touching, mounting, copulation and the whole mating sequence among mating pair combinations (touching: *F*_1,63_ = 2.05, mounting: *F*_1,62_ = 0.46, copulation: *F*_1,52_ = 0.35, whole mating sequence: *F*_1,66_ = 0.02 for all *p* > 0.05, Table 1). Additionally, the duration of mounting and copulation was not affected by the direction of mounting (mounting: *F*_4,62_ = 1.05, copulation: *F*_4,52_ = 0.47, for all *p* > 0.05). However, we found significant effects of the direction of approaching on the time length of touching when testing within each mating pair (*F*_3,63_ = 2.80, *p* = 0.0473, Table 2). Specifically, Mv–Fv displayed a longer touching time when approaching from the front compared to from the back (*p* = 0.05, Table 2), whereas mounting time involving left-side mounting behavior in Mexp–Fv lasted significantly longer than mounting behavior from the back (*p* = 0.0397, Table 3). The post-copula time between adults was affected by the sexual experience of males (*F*_1,66_ = 8.76, *p* = 0.0043, Table 1). In pair-wise comparisons, post-copula time in Mexp–Fexp lasted longer (mean ± SD: 55.8 ± 18.2 s) than in Mv–Fexp (mean ± SD: 9.3 ± 3.4 s, *p* = 0.0348, Table 1) but not when compared with Mexp–Fv or Mv–Fv (for all *p* > 0.05, Table 1). Post-copula was not affected by the sexual experience of the females (*F*_1,66_ = 0.70, *p* = 0.4063, Table 1) and the combined effect of sex and sexual experience (*F*_1,66_ = 0.70, *p* = 0.4048, Table 1).

## 4. Discussion

*Alphitobius diaperinus* exhibits lateralized or non-lateralized movements when approaching and mounting, which, together with sex and sexual experience, affects copulation success. According to previous research, the lateralized movements during the mating behavior of insects may be related to their anatomical asymmetries, gland localization, increased production of olfactory and tactile signals, as well as to the higher presence of sensory structures on the left- or right-side, head or antennae [15,16,24,25,26]. Some morphological asymmetries have been described for *A. diaperinus* in thorax-related structures [27]; however, the abdomen of *A. diaperinus* seems to be rather symmetrical as females and males have a secretion reservoir on the ventral apex of their abdomen [28]. This anatomical feature and the associated gland cells may be related to the predominant occurrence of non-laterality in approaching or mounting. Nevertheless, as lateralization in approaching and mounting is more recurrent among sexually experienced adult beetles, learning might be involved in the displayed behavior and the higher mating success. Furthermore, the predominance of male approaches from the females’ left-side suggests that in these beetles, specialized neural circuits would probably act in opposing directions leading to lateralized sensory-motor displays and improvements in short-distance sex recognition as shown in other insects [12,15,16]. Moreover, the different ways experienced adults approach might be due to changes in anatomy during the adult phase in *A. diaperinus* [29], as reported in other insects [30,31]. Further studies are needed to understand asymmetrical differences in responses based on sex entirely.

Our results are consistent with previous findings on mating behavioral asymmetries in other insects, including stored-product beetle pests, where males display left-side approaches and the mounting of potential mates, resulting in higher mating success than right-side approaches [8,9,13,32,33]. However, these lateralized behaviors can be modified by context-dependent factors, resulting, for instance, in right-side copulation approaches [6,7,10,12,16]. Non-lateralized behavior has also been observed in some insects, where males approach potential mates from the front and turn 180° from their left-biased, resulting in higher mating success than turning 180° from their right-biased [6,7,8,33]. Moreover, Romano et al. [12] showed that lateralization behavior to robotic sensory and motor signals is influenced by both sex and sexual status in the behavioral responses of the beetle *Prostephanus truncatus*. The lateralized and non-lateralized behaviors exhibited by *A. diaperinus* are shown to enhance efficiency in copulation, as well as flexibility and adaptability in response to changing environmental factors. It suggests that external factors such as sensory inputs or experience-based learning and memory may affect how beetles process and respond to signals, which in turn can influence their lateralized behavior [14,15,16,34].

Concerning copulation efficacy, this study has shown the impact of behavioral asymmetries on the duration of the mating sequence. The duration of mate recognition was significantly higher on the front than on the backside. Therefore, the Mv preference for approaching the female’s backside greatly affected the duration of touching, which also affected the copulation efficacy. This suggests that a stronger non-lateralization confers a benefit in terms of improved motor control [35,36] or problem-solving [1]. However, Mexp does not show a copulation preference for any bias. This suggests that, in this specific context, specific directions in the mounting behavior of Mexp confer a benefit in terms of having more mounting attempts and being successful; it also influences learning ability since Mv made fewer lateralized mounting mistakes. Thus, Mexp succeeded in all biases [35]. Similarly, male laterality behavior significantly affected the duration of mate recognition, mounting, copula, the whole mating sequence or a combination of these mating phases in *C. ferrugineus*, *R. dominica*, *S. oryzae*, *T. castaneum*, *T. confusum*, *T. granarium*, and *T. molitor* beetle pests, which may influence the development of large insect populations [6,7,8,9,10,13,37]. Additionally, sex, sexual experience and lateralization behavior significantly affected the duration of mate recognition in *P. truncatus* [12]. In addition, the higher time of post-copula shown by experienced mating pairs may be related to the males’ increasing ability to fertilize the females’ eggs [8,11,26]. In this sense, our results highlight that the transformation of sexual experience led to modifying the adult asymmetric mating response, thus leading to a higher mating efficiency.

## 5. Conclusions

In conclusion, our research contributes to understanding the behavioral asymmetries exhibited by *A. diaperinus* during mating phases. We have demonstrated that the sex and sexual condition of adults affect non-lateralized and lateralized mate recognition and mating patterns, resulting in higher copulation success. Experienced males with a higher degree of lateralization displayed a greater mating success rate. Furthermore, the duration of mate recognition and copulation efficacy were affected by behavioral asymmetries of approaching. The study highlights the impact of sensory inputs, experience-based learning, and memory on the beetles’ processing and response to signals, influencing their lateralized behavior. The elucidation of intricate dynamics relating to mate recognition and mating behaviors within this invasive pest will help to establish a solid groundwork for the implementation of more precise and efficacious control measures for this pest. These findings expand our knowledge of the mating behavior of *A. diaperinus* and provide insights into how distinct factors influence such behaviors. Further research is needed to fully understand the underlying mechanisms and evolutionary significance of these asymmetries to develop effective strategies to manage this pest.

## Figures and Tables

**Table 1:** Effect of sex and sexual experience of *Alphitobius diaperinus* adults on the total time of the whole mating sequence (in seconds).

Mating Pair	Precopula (s)		Copula (s)	Post-Copula (s)	Whole Mating Sequence (s)
	Touching (s)	Mounting (s)			
Mv–Fv	12.5 ± 8.7	6.7 ± 11.3	11.0 ± 9.6	19.9 ± 35.5 ab	50.2 ± 41.9
Mv–Fexp	48.9 ± 87.7	5.0 ± 5.3	13.3 ± 21.4	9.3 ± 15.8 b	76.5 ± 85.9
Mexp–Fv	15.7 ± 21.9	4.6 ± 8.7	14.5 ± 10.9	29.8 ± 56.5 ab	64.6 ± 83.3
Mexp–Fexp	16.3 ± 26.7	2.4 ± 2.1	18.1 ± 19.2	55.8 ± 74.9 a	92.6 ± 88.5
The mating pair: *F*, *p*	*F*_1,63_ = 2.05, 0.1568	*F*_1,62_ = 0.46, 0.4990	*F*_1,52_ = 0.35, 0.5562	*F*_1,66_ = 0.70, 0.4048	*F*_1,66_ = 0.02, 0.8945
Sexual experience of males: *F*, *p*	*F*_1,63_ = 1.96, 0.1663	*F*_1,63_ = 1.87, 0.1759	*F*_1,52_ = 0.02, 0.8974	*F*_1,66_ = 8.76, 0.0043 *	*F*_1,66_ = 1.40, 0.2411
Sexual experience of females: *F*, *p*	*F*_1,63_ = 1.33, 0.2528	*F*_1,63_ = 0.14, 0.7136	*F*_1,52_ = 0.35, 0.5554	*F*_1,66_ = 0.70, 0.4063	*F*_1,66_ = 1.07, 0.3044
Tested mating pair (*n* = *Mv–Fv*, *Mv–Fexp*, *Mexp–Fv*, *Mexp–Fexp*)	19 + 21 + 13 + 17 = 70				

Values are means followed by SD. Different letters within each column indicate significant differences in the time length of a specific mating phase among mating pair combinations; linear model, *p* < 0.05. Asterisks indicate significant effects of a specific variable on the time length in a mating phase; linear model, *p* < 0.05. Where no asterisks or letters exist, no significant differences were noted.

**Table 2:** Effect of the direction of approaching *Alphitobius diaperinus* adults on the duration of touching (in seconds).

Touching (s)				
Direction of Approaching	Mv–Fv	Mv–Fexp	Mexp–Fv	Mexp–Fexp
Backside (*Ba*)	9.2 ± 8.1 b	36.2 ± 63.1	16.6 ± 28.2	5.2 ± 3.6
Frontside (*Fa*)	16.2 ± 8.2 a	79.0 ± 126.2	16.5 ± 5.0	24.3 ± 35.3
Right-side (*Ra*)	-	3.0	-	16.0
Left-side (*La*)	-	-	5.0	11.0
Directionality: *F*, *p*	*F*_1,17_ = 4.41, 0.0500 *	*F*_2,18_ = 1.45, 0.2610	*F*_2,10_ = 0.81, 0.4701	*F*_3,13_ = 1.14, 0.3678
Tested interactions (*n* = *Ba* + *Fa* + *Ra* + *La*)	10 + 9 + 0 + 0 = 19	13 + 7 + 1 + 0 = 21	8 + 4 + 0 + 1 = 13	6 + 9 + 1 + 1 = 17

Values are means followed by SD. Different letters within each column indicate significant differences in touching time among directions of approaching within a mating pair combination; linear model, *p* < 0.05. Asterisks indicate significant effects of directionality on touching time in a mating pair combination; linear model, *p* < 0.05. Where no asterisks or letters exist, no significant differences were noted.

**Table 3:** Effect of the direction of mounting *Alphitobius diaperinus* adults on the duration of mounting (in seconds).

Mounting (s)				
Direction of Mounting	Mv–Fv	Mv–Fexp	Mexp–Fv	Mexp–Fexp
Backside (*Bm*)	8.3 ± 13.5	6.1 ± 7.2	1.3 ± 0.5 b	2.0 ± 0.8
Front-right (*FRm*)	-	3.5 ± 0.7	-	1.7 ± 0.6
Front-left (*FLm*)	23.0	4.0	-	-
Right-side (*Rm*)	1.8 ± 1.5	3.4 ± 1.9	2.3 ± 2.3 ab	2.7 ± 1.6
Left-side (*Lm*)	2.0 ± 1.0	5.8 ± 6.3	14.7 ± 15.8 a	3.0 ± 4.0
Directionality: *F*, *p*	*F*_3,15_ = 1.16, 0.3587	*F*_4,16_ = 0.08, 0.9872	*F*_2,10_ = 4.22, 0.0469 *	*F*_3,13_ = 0.17, 0.9130
Tested interactions (*n* = *Bm* + *FRm* + *FLm* + *Rm* + *Lm*)	11 + 0 + 1 + 4 + 3 = 19	8 + 2 + 1 + 5 + 5 = 21	7 + 0 + 0 + 3 + 3 = 13	4 + 3 + 0 + 6 + 4 = 17

Values are means followed by SD. Different letters within each column indicate significant differences in mounting time among directions of mounting within a mating pair combination; linear model, *p* < 0.05. Asterisks indicate significant effects of directionality on mounting time in a mating pair combination; linear model, *p* < 0.05. Where no asterisks exist, no significant differences were noted.

## Data Availability

All the data related to the research work are presented in the manuscript. Further details are available from the authors upon request.
